# Ubiquitin-proteasome system-mediated ubiquitination modification patterns and characterization of tumor microenvironment infiltration, stemness and cellular senescence in low-grade glioma

**DOI:** 10.18632/aging.204650

**Published:** 2023-04-11

**Authors:** Jun Gu, Lijun Pang, Donghua Yan, Chunming Wang, Yuekun Song, Zhengshuai Jin, Zhenwei Xu, Yuanqing Mao, Shengzhe Liu, Sheng Chen

**Affiliations:** 1 The Affiliated Jiangsu Shengze Hospital of Nanjing Medical University, Suzhou, Jiangsu, China

**Keywords:** ubiquitination modification, low-grade glioma, tumor microenvironment, cancer stem cell, cellular senescence

## Abstract

The Ubiquitin-proteasome system (UPS) performs a crucial role in immune activation and tumorigenesis. Nevertheless, the comprehensive role of the ubiquitin-proteasome system in the low-grade glioma (LGG) tumor microenvironment (TME) remains unknown. Ubiquitination modification patterns in LGG patients and corresponding characteristics of tumor immune traits, CSC stemness, and cellular senescence were evaluated via a comprehensive analysis of 20 ubiquitination modification regulators. For quantification of the ubiquitination modification status of individual patients, the UM-score was constructed and associated with TME characteristics, clinical features, cancer stem cell stemness, cellular senescence, prognosis, and immunotherapy efficacy. We identified that alterations in multiple ubiquitination regulators are linked to patient survival and the shaping of the tumor microenvironment. We found two different styles of ubiquitination modification in patients with low-grade glioma (immune-inflamed differentiation and immune-exclude dedifferentiation), characterized by high and low UM-score, and the two regulatory patterns of ubiquitination modification on immunity, stemness feature, and cellular senescence. We demonstrate that the UM-score could forecast the subtype of LGG, the immunologic infiltration traits, the biological process, the stemness feature, and the cellular senescence trait. Notably, the UM-score was related to immunotherapeutic efficacy, implying that modifying ubiquitination modification patterns by targeting ubiquitination modification regulators or ubiquitination modification pattern signature genes to reverse unfavorable TME properties will provide new insights into cancer immunotherapy. This research indicated that the ubiquitin-proteasome system is crucial in the formation of TME complexity and multiformity. The UM-score can determine ubiquitination modification status in individual patients, bringing about more personalized and effective immunotherapeutic tactics.

## INTRODUCTION

Ubiquitination is a significant post-translational modification that regulates the levels and activities of many proteins, as well as the cell cycle, cell proliferation, and DNA repair [1]. Ubiquitin, E1-activating enzymes, E2-conjugating enzymes, E3 ligases, deubiquitinating enzymes (DUBs), and the 26S proteasome make up the UPS [2]. Ubiquitin is a highly conserved modifying molecule consisting of 76 amino acids that ties and marks target substrates through a cascading process involving the E1, E2, and E3 enzymes. The marked substrate is then identified and destructed by the 26S proteasome complex [3]. Abnormal degradation or accumulation of tumor suppressor proteins and oncoproteins results in dysregulated cell proliferation, genomic instability, and oncogenesis [4]. Ubiquitinated modifications and their regulators' expression levels (including E1, E2, E3, and DUB) are frequently maladjusted in cancers, which is important for tumor growth, metastatic spread, as well as treatment failure [5].

Low-grade glioma (LGG) is a heterogeneous cancer that accounts for roughly 20% of intracranial tumors [6]. It has a much higher survival rate than glioblastoma (GBM), but it is more aggressive. Furthermore, LGG can develop into GBM, and the recurrence rate of low-grade glioma remains high despite various treatment options (e.g., surgical resection, adjuvant radiotherapy, temozolomide), owing to its unique tumor microenvironmental features and the presence of tumor stem cells [7, 8]. As a result, it is critical to develop therapeutic strategies that are tailored to the characteristics of the tumor microenvironment and stem cell properties. Notably, ubiquitination modifications may play a part in TME formation and glioma stem cell differentiation [9]. Therefore, the study of ubiquitination modifications may provide a new perspective for improving cancer therapy.

Immunotherapy, a revolutionary antitumor treatment, has brought light to cancer patients [6]. Several recent studies have found a link between ubiquitination modifications and immunotherapy. Inhibiting USP8 alters the tumor’s inflammatory microenvironment, which improves immunotherapeutic efficacy [10]. UBE2T promotes a variety of biological functions in CSCs (self-renewal, drug resistance, tumorigenicity, and metastatic ability), and UBE2T inhibition can regulate CSC-induced tumor recurrence and treatment resistance [11]. Furthermore, several studies have also found a link between ubiquitination modifications and common tumorigenic processes. As an oncogene, USP21 enhances CSC stemness by activating the Wnt pathway, resulting in tumor progression [12]. Tumorigenesis necessitates close cooperation between proto-oncogenes and oncogenes [13]. We have a limited understanding of ubiquitination regulation because researchers have so far focused solely on the particular roles of some ubiquitination regulators or specific cellular pathways. Therefore, a comprehensive investigation of the expression of ubiquitination-modified regulators in low-grade glioma is essential. A deep and ongoing analysis of tumor stratification based on ubiquitination modifications will yield a novel approach to tumor biology research.

Herein, we used genomic data from 1115 LGG samples to evaluate ubiquitination modification patterns and determine tumor immune microenvironment characteristics, stem cell properties, and cellular senescence in patients with different ubiquitination modification modes. We found two different ubiquitination modification modes (immune-inflamed differentiation patterns and immune-exclude dedifferentiation patterns). Moreover, to evaluate ubiquitination modifications in individual patients, we constructed a scoring system (UM-score) that was identified to correlate with clinical prognostic and molecular pathological parameters of LGG. This scoring system was also found to be capable of predicting immunotherapeutic responses. Finally, the pan-cancer analysis confirmed the close relationship among the UM-score, immune infiltration, and stemness.

## RESULTS

### Landscape of ubiquitination modification regulators in low-grade glioma

**Figure 1:**
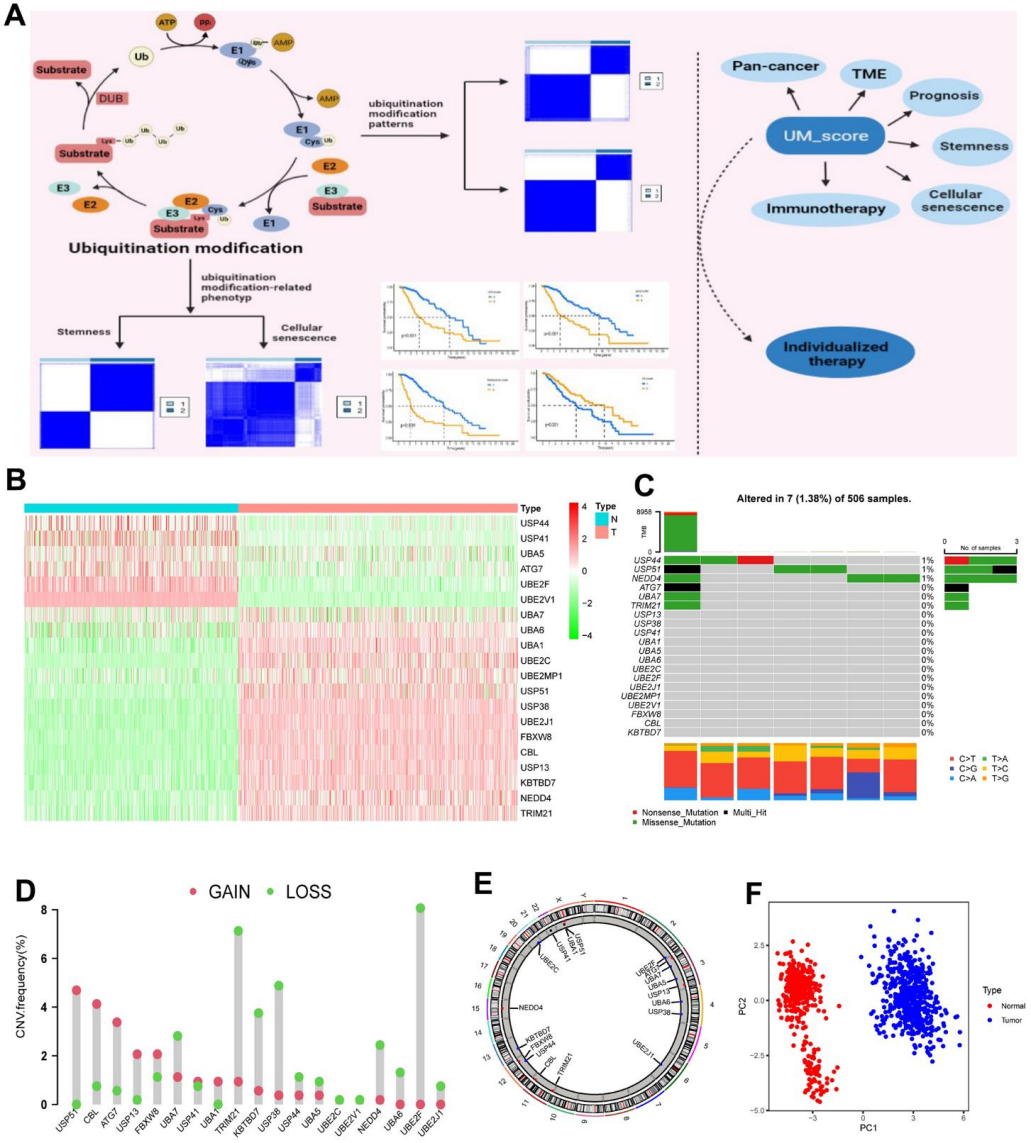
**Landscape of ubiquitination modification regulators in LGG.** (**A**) Graphic abstract of this research. (**B**) Differential expression of ubiquitination modification regulators. (**C**) Mutation occurrence of 20 ubiquitination modification regulators. (**D**) CNV alteration occurrence of ubiquitination modification regulators. (**E**) Chromosomal sites of altered CNV in the ubiquitination modification regulators. (**F**) Principal component analysis of the 20 ubiquitination modification regulators in normal and LGG patients.

Twenty ubiquitination regulators (5 E1 activating enzymes, 5 E2 conjugating enzymes, 5 E3 ligases, and 5 DUBs) were comprehensively investigated in this research. The entire design of this research is described in Supplementary Figure 1A. The dynamic, invertible course of ubiquitination modification adjusted by regulators as well as their underlying biological roles for proteins were depicted in Figure 1A. Differential expression analysis of 20 ubiquitination regulators revealed that all of these regulators were markedly different in normal vs. LGG samples (Normal=400, LGG=523; Figure 1B). We next described the occurrence of copy number variations and somatic mutations of 20 ubiquitination regulators in LGG. Among the 506 patients, the mutation occurrence of 20 ubiquitination regulators was 1.38% (7 mutations; Figure 1C). USP44 had the highest mutation occurrence, followed by USP51, whereas E2-conjugating enzymes had no mutations in LGG patients. Additional investigation exhibited a strong mutation co-occurrence correlation between UBA7 and ATG7, as well as TRIM21 and ATG7, along with TRIM21 and UBA7 (Supplementary Figure 1B). The analysis of CNV alteration occurrence revealed a widespread CNV alteration in the ubiquitination regulators (Figure 1D). Figure 1E depicts the site of CNV alterations in ubiquitination regulators on chromosomes. We were able to successfully differentiate LGG samples from normal samples depending on the expression of the 20 ubiquitination regulators (Figure 1F). To determine if the aforementioned genetic variations impacted the expression of ubiquitination regulators in LGG patients, we explored the correlation between ubiquitination regulator mRNA expression levels and CNV and discovered that the expression levels of most ubiquitination regulators positively correlated with their copy numbers (Supplementary Figure 1C). Tumorigenesis is a complicated process, and CNV changes cannot completely account for the differences in ubiquitination regulator expression. Other factors such as methylation and transcription can influence gene expression.

The above investigations showed remarkable heterology in the expression of ubiquitination regulators between normal and LGG samples, suggesting that an unbalance in the expression of ubiquitination regulators plays a critical role in the progression of LGG.

### Ubiquitination modification patterns mediated by 20 regulators

**Figure 2:**
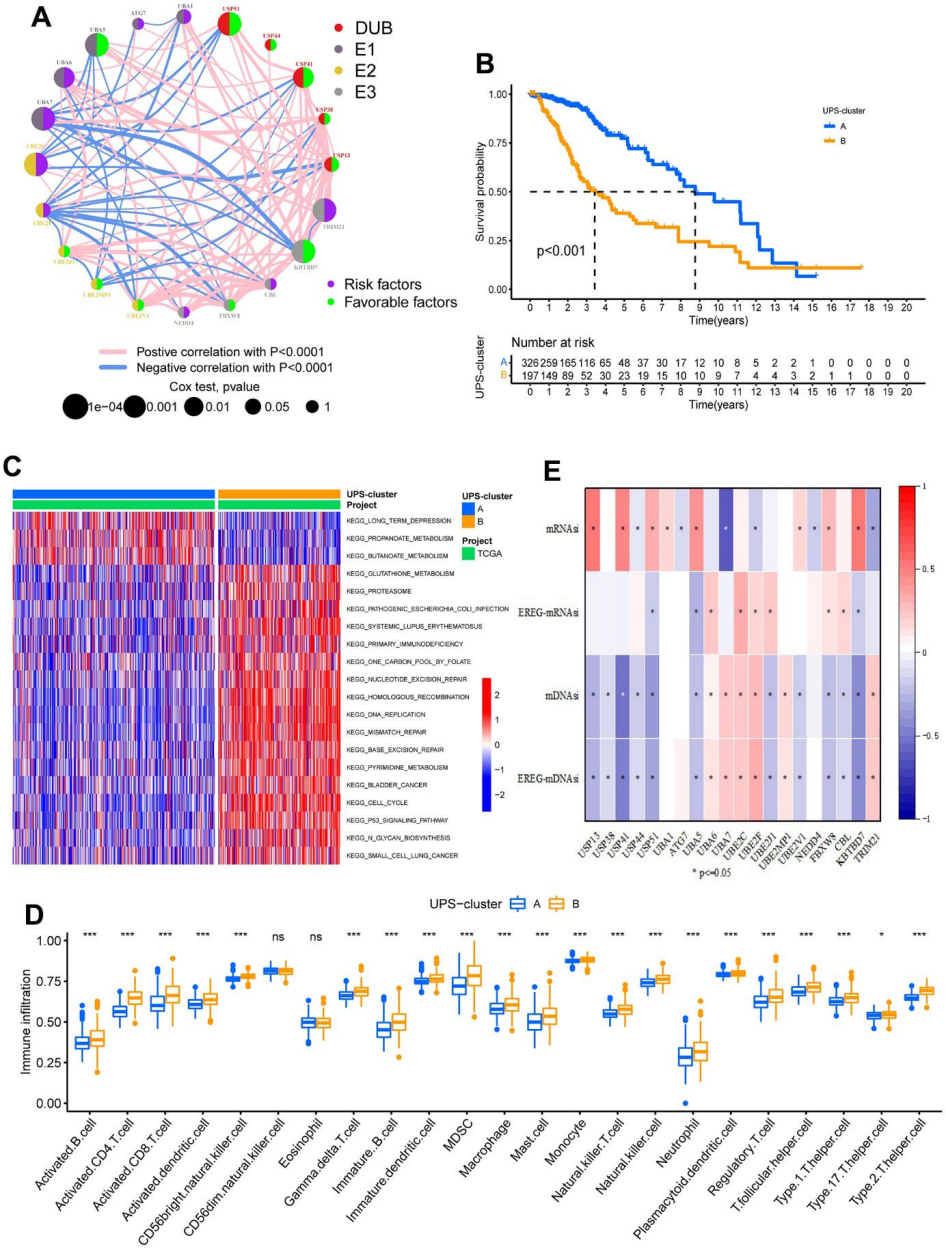
**Biological characteristics of two ubiquitination modification modes.** (**A**) The interplay between ubiquitination modification regulators in low-grade glioma. (**B**) Survival analyses for the two ubiquitination modification modes based on 523 patients with low-grade glioma from TCGA-LGG cohort. P<0.001. (**C**) GSVA analysis exhibits the activation states of biological pathways in two ubiquitination modification modes. (**D**) Abundance of Immune cell in the two ubiquitination modification modes. (**E**) The relationship between stemness index and the ubiquitination modification regulators.

Univariate Cox analysis confirmed the prognostic values of 20 ubiquitination regulators in low-grade glioma patients (Supplementary Figure 2A). The comprehensive landscape of ubiquitination regulator interactions and regulator relations, as well as the prognostic meaning for LGG patients, was illustrated with the ubiquitination regulator network (Figure 2A). To investigate the correlation among the regulators, we estimated pairwise connections between the expression of 20 ubiquitination regulators in LGG and discovered that positive associations outnumbered negative correlations (Supplementary Figure 2B). We discovered that not only the ubiquitination regulators in the homo-functional class exhibited a remarkable connection in expression, but there was also a strong relation between E1, E2, E3, and UDBs. Notably, we also observed that all ubiquitination regulators were significantly linked to immune cell infiltration and biological processes related to the regulation of the tumor microenvironment (Supplementary Figure 2C, 2D). Thus, cross-talk among the regulators of E1, E2, and E3 and UDBs could be crucial for the generation of distinct ubiquitination modification patterns and immune characteristics among LGG patients.

Next, we used consensus clustering to classify the ubiquitination modification patterns of LGG based on the expression levels of 20 ubiquitination regulators. After unsupervised clustering, 326 LGG patients were found in UPS-cluster A, while the remaining 197 patients were found in UPS-cluster B (Supplementary Figure 3A–3C and Supplementary Table 1). Survival analysis of the two ubiquitination modification patterns indicated that UPS-cluster A has a better prognosis (Figure 2B). Furthermore, the CGGA-LGG dataset (as the validation cohort) was used to conduct the clustering analysis with the same method to validate the efficacy of unsupervised clustering. Notably, the same outcomes were gained, demonstrating the efficacy of our clustering (Supplementary Figure 3D–3F). Notably, there was a clear distinction in transcriptome expression patterns of ubiquitination modification regulators between the USP-clusters in the TCGA-LGG and CGGA-LGG cohorts (Supplementary Figure 3G–3H).

### Different patterns of ubiquitination modification related to immune infiltration

To evaluate the biological behavior of the different ubiquitination modification patterns, we used GSVA analyses (Figure 2C). Samples in UPS-cluster A exhibited marked enrichment of pathways linked to propanoate metabolism and butanoate metabolism, while samples in UPS-cluster B showed significant enrichment of the cell cycle, nucleotide excision repair, DNA replication, and mismatch repair pathways.

Numerous investigations have demonstrated that ubiquitination modifications perform an essential role in the formation of TME and the DNA damage response [14, 15]. As a result, we first compared the differences in immune and stromal scores between ubiquitination modification patterns. The UPS-cluster B group had higher immune and stromal scores than the UPS-cluster A group (Supplementary Figure 4A, 4B). Subsequently, we identified distinctions in the DNA damage response-related biological processes among the ubiquitinated modification clusters. These biological processes, such as DNA damage repair, DNA replication, and mismatch repair, were markedly boosted in the UPS-cluster B group compared to the UPS-cluster A group (Supplementary Figure 4C). Furthermore, when comparing the UPS-cluster B group to the UPS-cluster A group, both pro- and anti-tumor immune signatures were upregulated, indicating that the ubiquitination modification pattern has a dual effect on anti-tumor immunity (Supplementary Figure 4D). The UPS-cluster B group had a higher abundance of immune cell infiltration compared to the UPS-cluster A group (Figure 2D). However, the UPS-cluster B group with higher immune cell infiltration had a worse prognosis. We speculated that the UPS-cluster B group’s DNA damage response-related phenotype suppressed immune cells’ anti-tumor capacity. Furthermore, the large amount of stroma in UPS-cluster B may confine immune cells around tumor cells to the periphery and prevent them from infiltrating into the tumor core, resulting in immune exclusion. Therefore, the UPS-cluster A group presented an immune-inflamed phenotype, and the UPS-cluster B group presented an immune-exclude phenotype. Given that biological processes like DNA damage repair and replication are linked to the CSC phenotypes and cellular senescence phenotypes, and the UPS plays a critical role in the regulation of the cell-cycle, we looked into the regulatory patterns of ubiquitination modification on the stemness feature and cellular senescence.

### Distinct patterns of ubiquitination modification associated with stemness feature

Mounting studies have shown that ubiquitination is essential for the self-renewal, maintenance, differentiation, and tumorigenesis of CSCs [16]. Stemness indices (si) describe the differentiation status of stem cells. We obtained four stemness indices (mRNAsi, EREG-mRNAsi, mDNAsi, and EREG-mDNAsi) from previous studies to comprehensively analyze the stemness characteristics of LGG stem cells. Further analysis revealed marked correlations between regulator expression and stemness indices (Figure 2E). Moreover, the stemness of cancer stem cells is significantly linked to the prognosis of LGG patients (Supplementary Figure 4E). Distinctions in stemness indices were observed between the two ubiquitination modification patterns; particularly, the dedifferentiation phenotype was most apparent in the UPS-cluster B, whereas the differentiation phenotype was most apparent in the UPS-cluster A (Supplementary Figure 4F).

**Figure 3:**
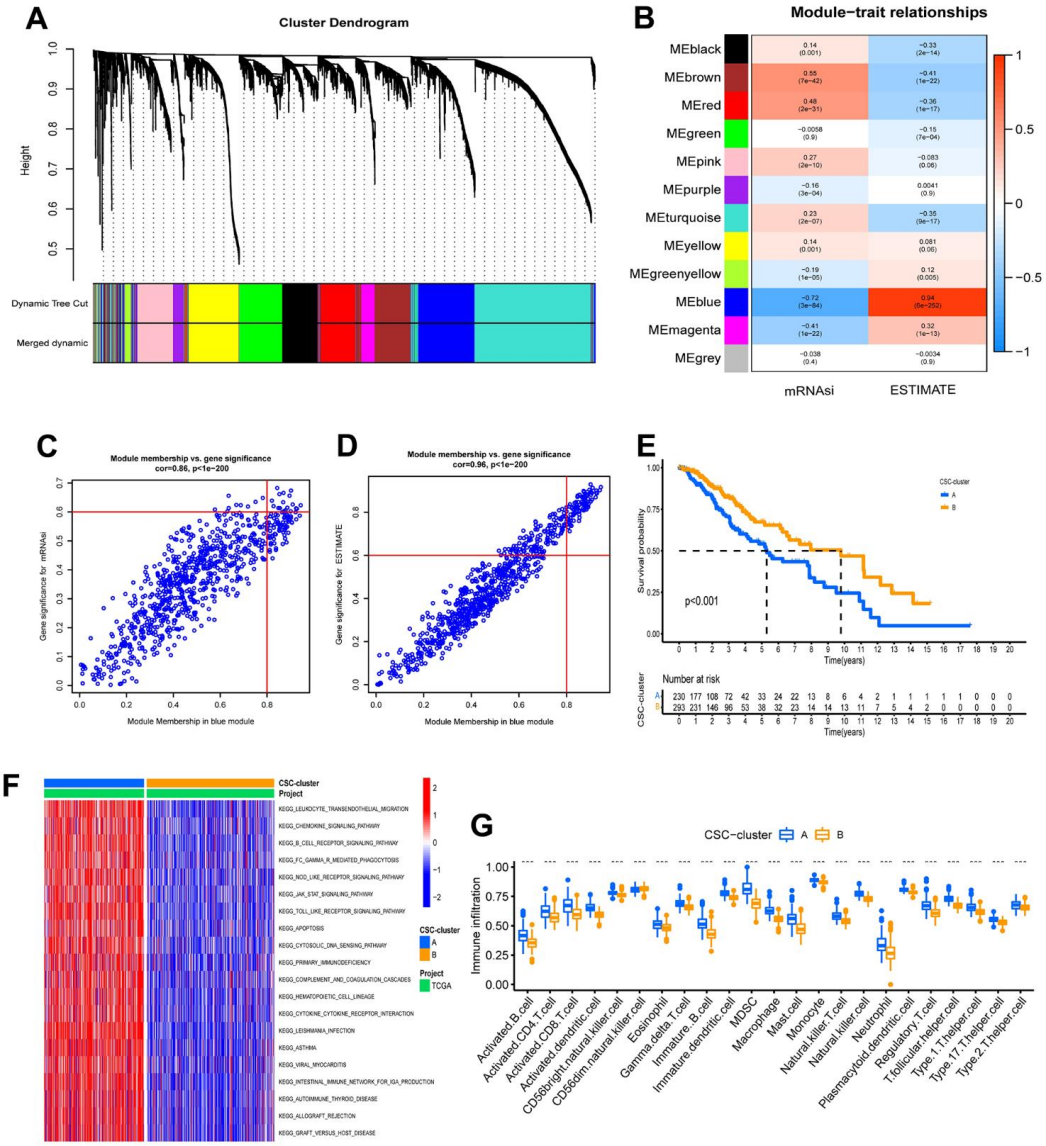
**Characteristics of ubiquitination modification-related phenotypes (CSC stemness).** (**A**) Hierarchical clustering dendrograms of identified co-expressed genes in modules. (**B**) Correlations between the gene modules and clinical traits. (**C**, **D**) The hub genes in blue modules. (**E**) Survival analyses for the two CSC-clusters based on 523 patients with low-grade glioma from TCGA-LGG cohort. (**F**) GSVA enrichment analysis in the two CSC-clusters. (**G**) Abundance of Immune cell in the two CSC-clusters.

To further investigate the correlation between cancer stem cell phenotype and TME in the two ubiquitination modification patterns, weighted correlation network analysis (WGCNA) was used to identify designated phenotype-related module genes. The phenotypes were defined using the ESTIMATE score and the mRNAsi. Differentially expressed genes (DEGs) were found using a cutoff criterion of |logFC| > 1 and P< 0.05, and these DEGs were used to construct a scale-free system. The scale-free plot indicated that 3 is the best parameter for transforming the adjacency matrix into a scale-free topology (Supplementary Figure 5A–5B). Finally, 12 modules (merged dynamic) were gained by merging modules whose distance was less than 0.25 (Figure 3A). The module Eigengenes were then determined, and the correlation between the module Eigengenes and the designated phenotypes was calculated (Figure 3B). Surprisingly, the blue module correlated the most with ESTIMATE scores and the mRNAsi index. Therefore, we chose hub genes in the blue module for further investigation and discovered 18 stemness phenotype-associated genes and 88 TME phenotype-associated genes (Figure 3C, 3D). Importantly, 18 of these two kinds of genes overlap, indicating that ubiquitination modifications have cross-reactivity with TME and stem cell regulation.

To explore the underlying biological behavior of the ubiquitination modification modes, we performed an unsupervised consensus clustering analysis of these 18 overlapped genes and further classified the samples into the corresponding cancer stem cell subtypes. As with the clustering results of the ubiquitination modification pattern, LGG patients were clustered into 2 distinct stem cell phenotypes (Supplementary Figure 5C–5E). Patients with CSC-cluster B had a better prognosis than patients with CSC-cluster A (Figure 3E). The expression patterns of ubiquitination modification regulators were significantly different in these two stem cell clusters, which again suggest that the two ubiquitination modification patterns have different effects on the tumor stem cell phenotype (Supplementary Figure 5F). The GSVA enrichment analysis showed immune-related biological processes such as leukocyte transendothelial migration, chemokine signaling pathways, B cell receptor signaling pathways, and cytokine-cytokine receptor interaction were significantly upregulated in the CSC-cluster A group (Figure 3F). Moreover, the CSC-cluster A group exhibited prominent enrichment of immune pathways (CD8 T effector, immune checkpoint, and antigen processing pathways) (Supplementary Figure 5G), as well as massive infiltration of immune cells (both pro- and anti-tumor immune cells) (Figure 3G), confirming again the intersectionality of ubiquitination modification regulating cancer stemness and immunity. The CSC-cluster B showed the opposite pattern (relatively few immune cells and weak enrichment of immune pathways). Importantly, the stromal-related pathways such as EMT and pan-fibroblast TGF response signaling pathways were also significantly enhanced in the CSC-cluster A, which can prevent the immune cell from infiltrating into the tumor core, resulting in a worse prognosis for the CSC-cluster A group.

### Distinct patterns of ubiquitination modification associated with cellular senescence

**Figure 4:**
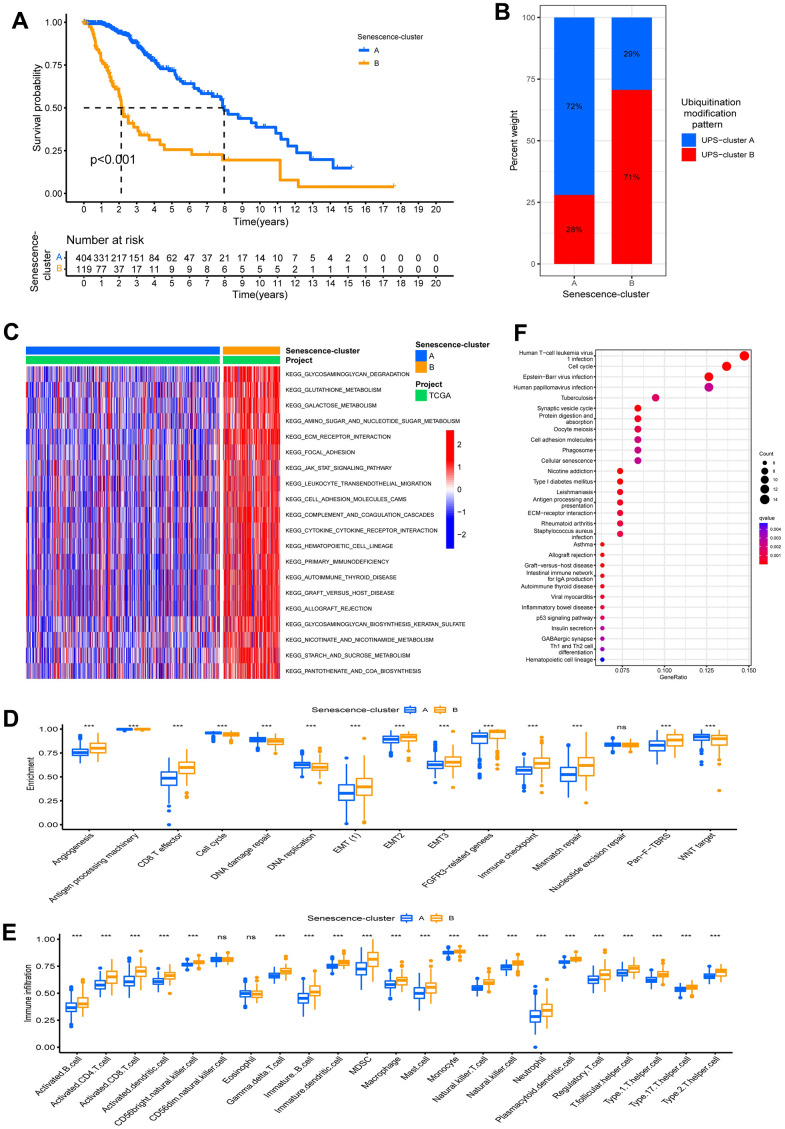
**Characteristics of ubiquitination modification-related phenotypes (cellular senescence).** (**A**) Survival analyses for the Senescence-clusters based on 523 patients with low-grade glioma from TCGA-LGG cohort. (**B**) The different ubiquitination modification patterns distribution between the Senescence-clusters. (**C**) GSVA analysis in the Senescence-clusters. (**D**) Enrichment score of biological process in the Senescence-clusters. (**E**) Abundance of Immune cell in the Senescence- clusters. (**F**) KEGG enrichment analysis of the differential expression genes between UPS- clusters.

Cellular senescence is a stress response induced by damage, which can result in the release of multiple cytokines, chemokines, and proteinases, ultimately leading to tumor immune microenvironment remodeling [17]. To further investigate the correlation between the cellular senescence phenotype and TME in the two ubiquitination modification patterns, we obtained 278 cellular senescence genes from CellAge and then 270 differentially expressed cellular senescence genes by differential analysis (p <0.05). Surprisingly, similar to the ubiquitination modification pattern clustering results, unsupervised clustering analysis based on these 270 cellular senescence-associated genes clustered LGG patients into two distinct cellular senescence phenotypes (Senescence-cluster A and B; Supplementary Figure 6A–6C). Patients with senescence cluster A had a better prognosis than patients with senescence cluster B (Figure 4A). The expression modes of ubiquitination modification regulators were greatly distinct in the two senescence clusters (Supplementary Figure 6D). Furthermore, patients in cellular senescence cluster A predominantly exhibited ubiquitination modification pattern A, whereas patients in cellular senescence cluster B predominantly exhibited ubiquitination modification pattern B, which suggests that the two ubiquitination modification patterns have different effects on the tumor cellular senescence phenotype (Figure 4B).

The GSVA enrichment analysis showed that the immune-related biological processes such as leukocyte transendothelial migration and cytokine-cytokine receptor interaction and stromal-related pathways like ECM receptor interactions were significantly upregulated in the Senescence-cluster B group, whereas they were relatively decreased in Senescence-cluster A (Figure 4C). We further identified differences in the enrichment of stromal-related biological processes among the cellular senescence phenotypes. Stromal-related biological processes such as angiogenesis, EMT, and Pan−F−TBRS were markedly increased in the senescence-cluster B group compared to the senescence-cluster A group. Moreover, the Senescence-cluster B group exhibited prominent enrichment of immune pathways (CD8 T effector, immune checkpoint, and antigen processing pathways) as well as abundant infiltration of immune cells (both pro- and anti-tumor immune cells), confirming the intersectionality of ubiquitination modification regulating cellular senescence and immunity (Figure 4D, 4E). The Senescence-cluster A displayed the inverse trend (relatively few immune cells and weak enrichment of immune pathways).

### Construction of ubiquitination modification signature

To verify the underlying biological course of the 2 ubiquitination modification modes, we recognized 216 DEGs linked to the ubiquitination modification modes using the limma package (Supplementary Figure 6E). KEGG analysis of DEGs revealed enrichment mainly in cell cycle, cellular senescence, cell adhesion molecules, ECM receptor interactions, and antigen processing and presentation pathways, confirming that ubiquitinated modifications regulate the tumor microenvironment through tumor stem cell phenotype and cellular senescence phenotype (Figure 4F). To confirm the adjustment mechanism of ubiquitination modification on the TME, we conducted the Lasso method on 216 DEGs to gain 37 signature genes of ubiquitination modification mode and then classified patients into distinct gene clusters relying on an unsupervised cluster algorithm of these genes (Supplementary Figure 7A). Aligned with the clustering results of ubiquitination modification regulators, the unsupervised clustering based on signature genes divided LGG patients into 2 classifications, which were dubbed UPS gene clusters A and B (Supplementary Figure 7B–7D). This outcome proved that LGG does have two distinct ubiquitination modification modes. To confirm the relationship of ubiquitination modification modes to the TME, we investigated the enrichment level of common TME signatures in the UPS gene clusters. Gene-cluster B had markedly increased stromal activity, as demonstrated by angiogenesis and upregulation of EMT signatures (Supplementary Figure 7E). Meanwhile, both pro- and anti-tumor immune signatures were abundant in UPS gene cluster B (Supplementary Figure 7F). Similarly, the patients belonging to gene cluster A (immune-inflamed phenotype) had a better prognosis (Supplementary Figure 7G). These outcomes were consistent with previous analysis and showed the importance of ubiquitination modifications in shaping the TME of LGG, which can categorize LGG patients as having an immune-inflamed phenotype or an immune-exclude phenotype.

**Figure 5:**
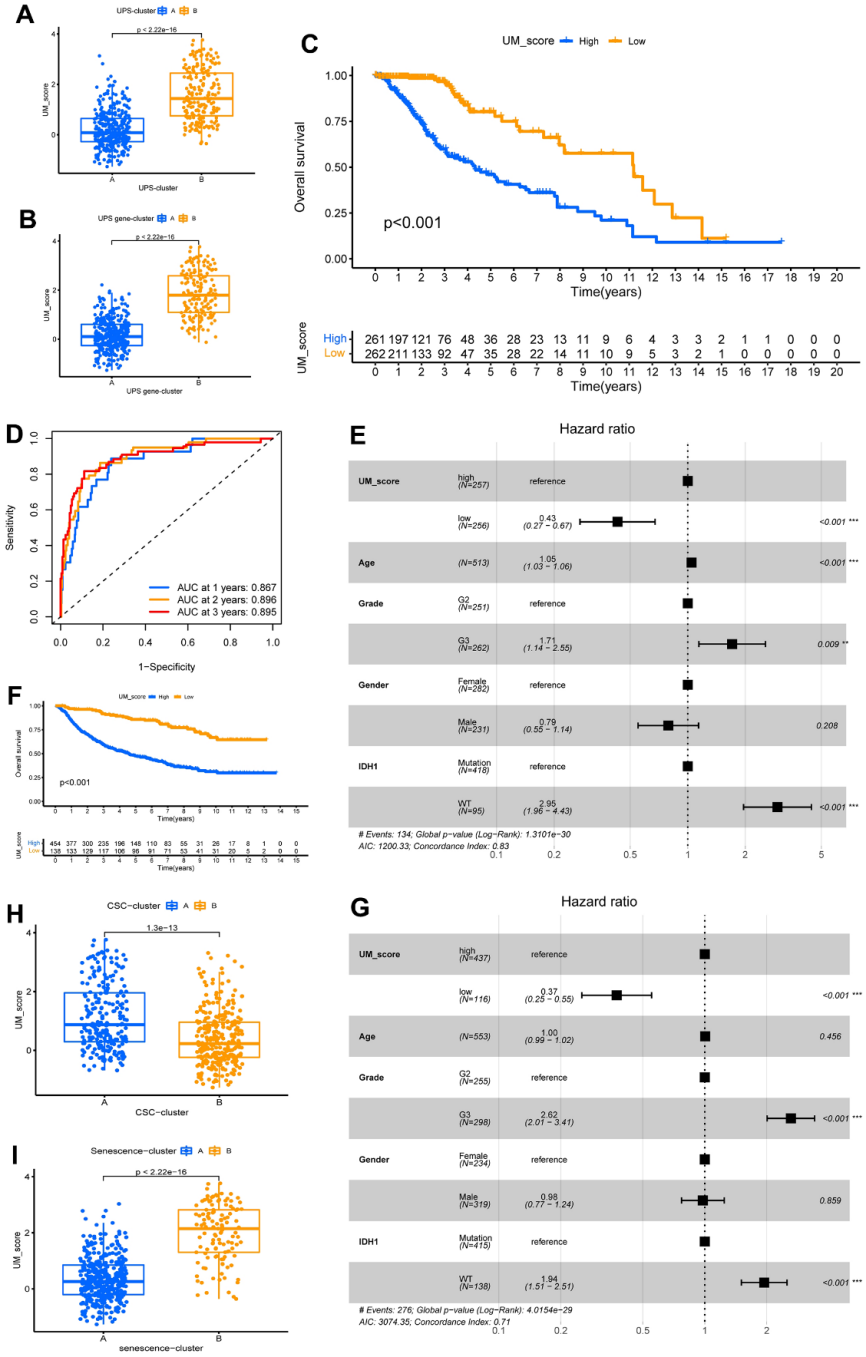
**Construction of ubiquitination modification characteristic signature.** (**A**, **B**) Distinction in the UM-score between ubiquitination modification modes and gene clusters in TCGA-LGG cohorts. (**C**) Survival analyses for the UM-score groups based on 523 patients with low-grade glioma from TCGA-LGG cohort. (**D**) The forecast value of UM-score in patients among the TCGA-LGG cohorts (AUC: 0.867, 0.896, and 0.895; 1, 2, and 3-years overall survival). (**E**) Multivariate Cox regression analysis, which included the factors of UM-score, Age, Grade, Gender, IDH1 status and patient outcomes in the TCGA-LGG cohort. (**F**) Survival analyses for the high- and low-UM-score groups based on 592 patients with low-grade glioma from CGGA-LGG cohort. (**G**) Multivariate Cox regression analysis, which included the factors of UM-score, Age, Grade, Gender, IDH1 status and patient outcomes in the CGGA-LGG cohort. (**H**, **I**) Distinction in the UM-score between CSC-clusters and Senescence-clusters in TCGA-LGG cohorts.

In consideration of the heterogeneity and complexity of ubiquitination modifications, we needed to be able to accurately evaluate ubiquitination modification modes in individual LGG patients. We identified 7 independent prognosis-related signature genes using a multivariate Cox analysis of 37 ubiquitination modification mode signature genes (Supplementary Table 2). We then created a prognosis-related signature gene-based score system by Lasso analysis to assess ubiquitination modification patterns in individual patients with LGG, and Supplementary Table 3 shows 6 genes used to construct the score and their coefficients; the model was dubbed the UM-score (Supplementary Table 4). We discovered the UM-score of the UPS-cluster B group was considerably higher than that of the UPS-cluster A group (Figure 5A). In consistence, gene-cluster B had a considerably higher UM-score than gene-cluster A (Figure 5B). To evaluate the clinical significance of the UM-score further, we split patients into high- and low-UM-score subgroups using the media score. Patients with a low UM-score had a significant survival advantage (Figure 5C).

The AUCs of the ROC analysis for the UM-score were 0.867, 0.896, and 0.895 at 1, 2, and 3 years of OS, respectively (Figure 5D). We used the multivariate Cox method with patient clinical features such as age, gender, grade, and the IDH1 mutation to see if the UM score could serve as an independent prognostic factor. We discovered that the UM-score was a reliable and independent prognostic biomarker for assessing patient outcomes (Figure 5E; HR =0.43, 95% CI 0.27–0.67, *p<*0.001). The dependability of the UM-score was validated utilizing 553 CGGA-LGG patients. Aligned with above results, the low-UM-score group had a better prognosis than the high-UM-score group (Figure 5F) and multivariate Cox method also affirmed that the UM-score could be used as an independent prognosis factor (Figure 5G; HR = 0.37, 95% CI 0.25–0.55, *p<*0.001). These findings suggest that the UM-score can assess the ubiquitination modification modes and forecast the prognosis of LGG patients.

### The role of the UM-score in assessing tumor microenvironment and immunotherapy

**Figure 6:**
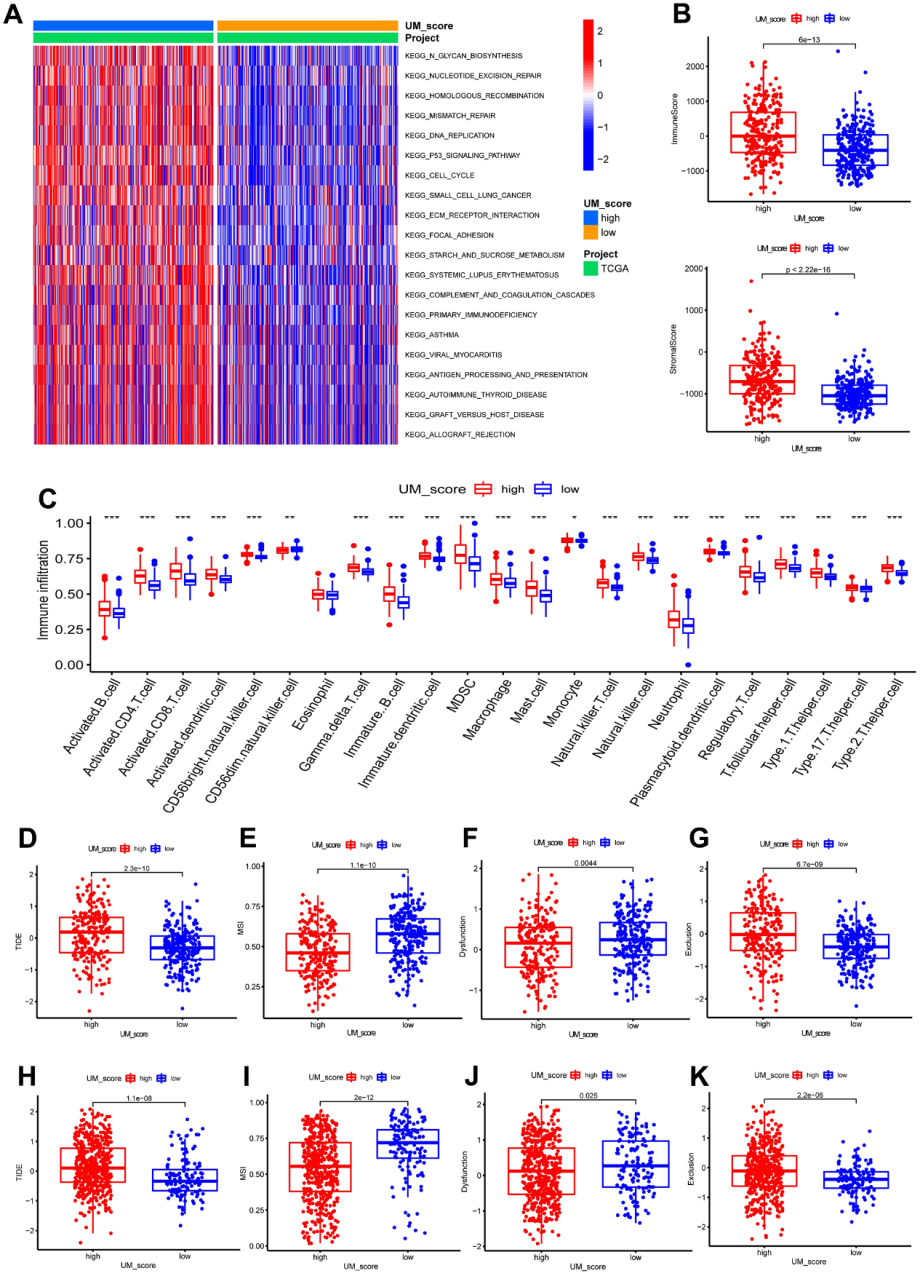
**UM-score in the role of immunotherapy.** (**A**) GSVA enrichment analysis in the UM-score subgroups. (**B**) Distinction in the immune scores and stromal scores between the UM-score subgroups in TCGA-LGG cohorts. (**C**) Abundance of immune cell in the UM-score subgroups. (**D**) Distinction in the TIDE score between the UM-score subgroups in TCGA-LGG cohorts. (**E**) Distinction in the MSI score between the UM-score subgroups in TCGA-LGG cohorts. (**F**) Distinction in the T-cell dysfunction score between the UM-score subgroups in TCGA-LGG cohorts. (**G**) Distinction in the T-cell exclusion score between the UM-score subgroups in TCGA-LGG cohorts. (**H**) Distinction in the TIDE score between the UM-score subgroups in CGGA-LGG cohorts. (**I**) Distinction in the MSI score between the UM-score subgroups in CGGA-LGG cohorts. (**J**) Distinction in the T-cell dysfunction score between the UM-score subgroups in CGGA-LGG cohorts. (**K**) Distinction in the T-cell exclusion score between the UM-score subgroups in CGGA-LGG cohorts.

We further analyzed the correlation between UM-score and the two ubiquitination modification-related phenotypes. We discovered that UM-score of CSC-cluster A was markedly higher than CSC-cluster B (Figure 5H). Moreover, Senescence−cluster B had markedly higher UM-score than Senescence−cluster A (Figure 5I). Given the two ubiquitination modification-related phenotypes play a significant role in tumor immune microenvironment, we speculated the UM-score could be used to assess the immunal traits in patients with LGG. To explore the biological traits difference between UM-score groups, we used GSVA analysis on the two groups (Figure 6A). Samples in the high-UM-score group exhibited markedly enrichment of the cell cycle, nucleotide excision repair, DNA replication as well as mismatch repair pathways, whereas the samples in the low-UM-score group were on the contrary. The high-UM-score group had higher immune activity and stroma activity (Figure 6B and Supplementary Table 5), and the UM-score was considerably positively linked to immune scores (Supplementary Figure 8A; R = 0.42, p < 2.2e-16) as well as stroma scores (Supplementary Figure 8B; R = 0.46, p < 2.2e-16). Stromal-related biological processes such as angiogenesis, EMT and Pan−F−TBRS were significantly enhanced in high-UM-score group compared to low-UM-score group (Supplementary Figure 8C). Moreover, the high-UM-score group exhibited prominent enrichment of immune pathways (CD8 T effector, immune checkpoint, and antigen processing machinery) as well as substantial infiltration of immune cells (both pro- and anti-tumor immune cells; Figure 6C). While the low-UM-score subtype patients had a higher enrichment of Fibroblast Growth Factor Receptor 3 (FGFR3), the RTK/RAS pathway, and the PI3K pathway (Supplementary Figure 8D). These findings indicate that the UM-score can evaluate the tumor immune microenvironmental traits of LGG patients.

Given that the expression of most immune checkpoints is significantly enhanced in the high-UM-score group, we speculated that the UM-score could be used to assess the immunotherapy responses in patients with LGG. TIDE scores have been proven to accurately predict the immunotherapeutic response. Patients with higher TIDE scores had a poorer immunotherapeutic response, implying immunotherapy was less likely to benefit them. In this study, TIDE scores were lower in patients with a low UM score, suggesting that ICI treatment was more effective on patients in the low UM score group (Figure 6D). To identify the source of the distinctions in immunotherapeutic response, we compared microsatellite instability (MSI), T-cell exclusion, and T-cell dysfunction scores between the UM-score groups (Supplementary Table 6). The low-UM-score group had higher scores for MSI and T-cell dysfunction, whereas the high-UM-score group had higher scores for T-cell exclusion (Figure 6E–6G). These results were verified by the CGGA cohort (Figure 6H–6K).

### The utility of the UM-score across cancer types

Due to the previously mentioned close connection between UM-score and immunotherapy response, we subsequently investigated the role of the UM-score in various cancers. We discovered that the UM-score was linked to the prognosis of multiple kinds of cancer, as shown in Supplementary Figure 9A. Notably, UM-score had the highest HR on LGG prognosis, implying that ubiquitination modifications perform essential parts in the progression of low-grade glioma. MSI and TMB were meaningful biomarkers for immune checkpoint blockade therapy response. Radar plots revealed a significant correlation between UM-score and TMB in 12 of 33 cancers (Supplementary Figure 9B). Subsequently, we looked at the correlation between MSI and UM-score and discovered that ESCA had the strongest negative correlation (Supplementary Figure 9C). Supplementary Figure 9D shows that the levels of PD-L1 expression were significantly related to the UM-score. These findings confirm the scoring system’s ability to accurately predict immunotherapy response. Furthermore, we discovered a different relationship between the three biomarkers and UM-score in some cancer types. This phenomenon could be explained by the fact that cancers differ in terms of immune infiltration. We further investigated the relationship between the content of 22 immune cells and the UM-score, and discovered that the ratio of M1 to M2 macrophages correlated with the UM-score of the majority of cancer types (Supplementary Figure 9E). Furthermore, with the exception of ACC, CESC, DLBC, ESCA, KICH, OV, THYM and UCS, we discovered a link between UM-score and stemness index in 24 cancers (Supplementary Figure 9F).

All these results indicate that ubiquitination modifications play an indispensable role in shaping the tumor microenvironment.

## DISCUSSION

Growing studies suggest that ubiquitination modifications perform a vital role in governing cell cycle, DNA repair, signal transduction, immunity, and antitumor activity through interaction between various ubiquitination modification regulators (E1 activating enzymes, E2 conjugating enzymes, E3 ligases, and DUBs) [18]. Whereas most research has concentrated on a single type of ubiquitination modification regulator, the mutual connections and roles of multiple kinds of regulators in cancer remain unknown. We discovered the comprehensive alterations of E1, E2, E3 and DUBs at transcription and genetic levels and their mutual association in LGG. Then we confirmed two different ubiquitination modification patterns depending on the expression of 20 ubiquitination modification regulators and got a greater understanding of the tumor microenvironment with distinct modification modes. Further investigation of the stemness indices determined the features of the modification modes: the UPS-cluster A group exhibited an immune-inflamed differentiation phenotype, and the UPS-cluster B group exhibited an immune-exclude dedifferentiation phenotype. Unsupervised clustering methods of the hub genes identified by WGCNA revealed the underlying mechanisms by which ubiquitination modifications regulate the immune and stemness phenotypes. Furthermore, we also performed unsupervised clustering of the cellular senescence genes to determine the cellular senescence subgroups and revealed the intersectionality of ubiquitination modification regulating cellular senescence and immunity. Ultimately, we established a scoring system, the UM-score, to assess ubiquitination modification patterns in individual patients, providing a clinical tool for a more individualized and effective immunotherapy strategy. The patients with the lower UM-score have a better prognosis. The abundance of immune cells in the TME is markedly distinct between the two LGG groups, and a high UM-score group is linked to higher infiltration of immune cells (both pro- and anti-tumor immune cells). This LGG group is also characterized by a substantial activation of angiogenesis, EMT, and Pan-F-TBRS signaling pathways, which prevent the immune cell from infiltrating into the tumor core, resulting in a worse prognosis.

Emerging functions of ubiquitination modification in CSCs, cellular senescence, and immune infiltration have been affirmed, including roles in tumor metabolism regulation, tumor immune microenvironment modulation, cancer stem cell stemness maintenance, and cellular senescence induction [19]. Schimmer et al. proved that inhibiting the E1-activating enzyme UBA1 reduced leukemia burden and specifically targeted leukemia stem cells [20]; Ma et al. demonstrated that the E2-conjugating enzyme UBE2B promoted ovarian cancer growth [21]; Wang et al. discovered that deleting the E3 ligases TRIM29 inhibited PDAC’s cancer stem cell-like properties by accelerating ISG15 degradation [22]; Lee et al. revealed that USP1-mediated protein stabilisation promoted GSC maintenance and treatment resistance [23]. Besides, targeting E3 ligase Skp2 attenuates aerobic glycolysis and induces cellular senescence in cancer cells, thereby reducing CSC populations and their function [24]. These results suggest that additional comprehensive research is required to verify the expression and function of ubiquitination modification regulators in LGG. As most studies focused on the features of a specific regulator, our insight into the TME, stemness traits, and cellular senescence regulated by ubiquitination modification regulators is limited. Discovering the link among the ubiquitination modification patterns in TME, cancer stemness, and cellular senescence of LGG will help to expand our insight into ubiquitination modification and develop a more effective treatment strategy.

To our knowledge, this is the first research to develop a strategy for assessing the immune, stemness features, and cellular senescence of ubiquitination modification patterns of LGG and to quantify modification patterns using a machine-learning algorithm. Recent research has revealed unusual connections among ubiquitination modification and immune infiltration, cancer stem cells, cellular senescence, and treatment resistance. USP6 exerts antitumor effects via increasing intra-tumoral chemokine production as well as the infiltration and activation of NK cells, dendritic cells, and macrophages [25]. Specific downregulation of UBA6 expression in T cells results in increased interferon-gamma production, which in turn regulates T cell differentiation [26]. E3 ligase FBXO3 promotes ubiquitination of PD-1 of T cells, thereby enhancing anti-tumor immunity [27]. Targeting USP44 can reduce FOXP3 expression at the protein level, and thus break immune tolerance in tumor patients [28]. Moreover, knockdown of E3 ligase E6AP can inhibit tumor cell growth by promoting cellular senescence and enhances the sensitivity of tumor cells to radiation [29]. Significantly, USP9X is involved in maintaining the stemness maintenance of glioblastoma stem cells [30]. Herein, we discovered that the ubiquitination modification patterns were linked to various characteristics, including immune cell infiltration features, biological behavior, differentiation phenotypes, and cellular senescence. Researchers’ exact mechanistic studies of ubiquitination modification regulators, combined with our comprehensive macro-level analysis, may serve as a stepping stone for ubiquitination modification targeted therapy and improve TME. Notably, the high incidence of co-occurring mutations in regulators proves that combination treatment may be more effective than monotherapy.

Meaningfully, the high-UM-score group showed a significant increase in the EMT, angiogenesis, TGF- pathway, treg cells, and immune checkpoints when compared to the low-UM-score group, which can be the causes of immune suppression. The EMT is critical for tumor progression, metastasis, and drug resistance [31]. Treg cells suppress anti-cancer immunity, preventing an effective anti-tumor immune response in the tumor-bearing host and promoting tumor development [32]. Previous research has shown that Treg cell infiltration has been linked to tumor EMT and tumor cell invasion [33]. Yun et al. discovered that Treg cells can increase tumor cell TGF-β signaling and promote EMT, which result in metastasis [34]. The TGF-β signaling pathway is important in cell proliferation, EMT, and immune function suppression [35]. As a result, the abundant infiltration of Treg cells in the high-UM-score group may facilitate tumor EMT by activating the TGF-signaling pathway, and these alterations may increase angiogenesis in the TME, enhancing LGG invasion and metastasis. While the patients with low-UM-score had prominently longer survival and a higher enrichment of Fibroblast Growth Factor Receptor 3(FGFR3), RTK/RAS pathway, and PI3K pathway. FGFRs signaling regulates several biological functions, including cell proliferation, neural stem cell self-renewal, and progression of glioblastoma (GBM) [36]. Several studies have been carried out to look into the possibility of FGFR3 as a new therapeutic target [37]. Besides, activation of the RTK/RAS/PI3K/AKT signaling pathway has been shown to be a risk factor for GBM [38]. Therefore, FGFR3 and RTK/Ras/PI3K/AKT signaling pathways may perform a crucial part in affecting the prognosis of LGG patients with low-UM-score.

Cancer immunotherapy has emerged as an appealing cancer treatment, with one of the most remarkable achievements in cancer immunotherapy being the use of immune checkpoint inhibitors [5]. The main co-suppressive checkpoint pathway regulating immune escape in cancer patients is PD-1/PD-L1, and its activation and inhibition significantly alter the landscape of tumor clearance [39]. However, immune checkpoint inhibitors do not have stable efficacy in LGG patients. Ubiquitination and deubiquitination of PD-1/PD-L1 proteins are thought to perform a significant part in the stabilization and kinetic regulation of PD-1 and PD-L1 proteins [40]. Inspiringly, the UM-score can be used not only to quantify the pattern of ubiquitination modifications in individual patients but also to assess immunotherapy response. Patients in the high-UM-score group had higher TIDE and T-cell exclusion scores, implying that their lower immunotherapy response could be due to immune evasion caused by T-cell exclusion. The presence of a large amount of stromal in the high-UM-score group is a major cause of immune exclusion. The low-UM-score subgroup had higher MSI scores and T-cell dysfunction scores than the high-UM-score subgroup. MSI-caused high mutation rates have been proven to make cancer immunogenic and sensitive to ICI treatment [41]. Previous research has shown that dysfunctional T cells are not completely inactive, and that blocking immune checkpoints with anti-PD-1 monoclonal antibodies can successfully restore T cell function [42]. As a result, patients in the low-UM-score subgroup respond well to immunotherapy. According to the above findings, patients in different UM-score groups should receive different treatment strategies. More importantly, our findings indicate that modifying ubiquitination modification patterns by targeting ubiquitination modification regulators or ubiquitination modification pattern signature genes to reverse unfavorable cellular infiltration properties will provide new insights into cancer immunotherapy. It might contribute to the development of novel drug combinations or immunotherapeutic agents. Our study suggests novel approaches for enhancing patients’ clinical responses to immunotherapy, identifying unique tumor immune phenotypes, and promoting tumor-specific therapy.

## MATERIALS AND METHODS

### Data collection

Low-grade glioma transcriptome data were obtained from TCGA-LGG dataset. The RNAseq transcriptome data for normal tissues were gained from GTEx. CNV data were gained from Xena. CGGA-LGG transcription data were downloaded from the CGGA website for validation purposes. 119 ubiquitination modification regulators (Supplementary Table 7) were obtained from previous studies [3, 43–46]. Based on the differential analysis of the 119 ubiquitination modification regulators, we respectively selected the top 5 ranked E1, top 5 ranked E2, top 5 ranked E3 and top 5 ranked DUB based on |Log_2_FC| for further analysis.

### Unsupervised consensus clustering on LGG patients

Unsupervised clustering method was performed to recognize the ubiquitination modes as well as the phenotypes associated with these patterns (CSC stemness and cellular senescence).

### Gene set variation analysis

In non-parametric and unsupervised methods, GSVA was performed to assess the biological behaviour difference in distinct LGG patient subgroups (UPS-clusters, CSC-clusters, Senescence-clusters and UM-score subgroups).

### Evaluation of immune infiltration features

The ssGSEA method was performed to calculate relative abundances of immune cell per sample. The genomic information used to label immune cell subtype was gained from Charoentong’s research, which annotated human immune cell subtypes.

### Construction of LGG ubiquitination modification score (UM-score)

Among 37 ubiquitination modifications pattern signature genes, multivariate Cox method was performed to confirm 7 independent prognosis-related signature genes, which we selected for the development of a scoring system with the LASSO regression method. Ultimately, six genes and their coefficients were confirmed by the minimum criteria. The UM-score was calculated using the formula:


$$\text{UM-score}=\Sigma_{\text{i}=1}^{\text{n}}\;{\text{Coef}}_{\text{i}}*{\text{x}}_{\text{i}}$$

### Statistical analysis

The limma R package was applied to identify DEGs. The Spearman method was applied to conduct correlation analysis. The Wilcoxon rank sum test was applied to calculate the statistical difference between the 2 clusters. R tool was applied for all statistical tests.

### Availability of data and materials

The data used in the study are described in detail in “Data collection and processing”.

## Supplementary Material

Supplementary Figures

Supplementary Table 1

Supplementary Tables 2 and 3

Supplementary Table 4

Supplementary Table 5

Supplementary Table 6

Supplementary Table 7
