# Supplementary material for: Ubiquitin-proteasome system-mediated ubiquitination modification patterns and characterization of tumor microenvironment infiltration, stemness and cellular senescence in low-grade glioma
Source: Aging (Albany NY). 2023 Apr 11;15(8):2970–98. doi: 10.18632/aging.204650 (PMC10188348; doi:10.18632/aging.204650)
Supplement: Supplementary Figures [file aging-15-204650-s001.pdf]

SUPPLEMENTARY FIGURES

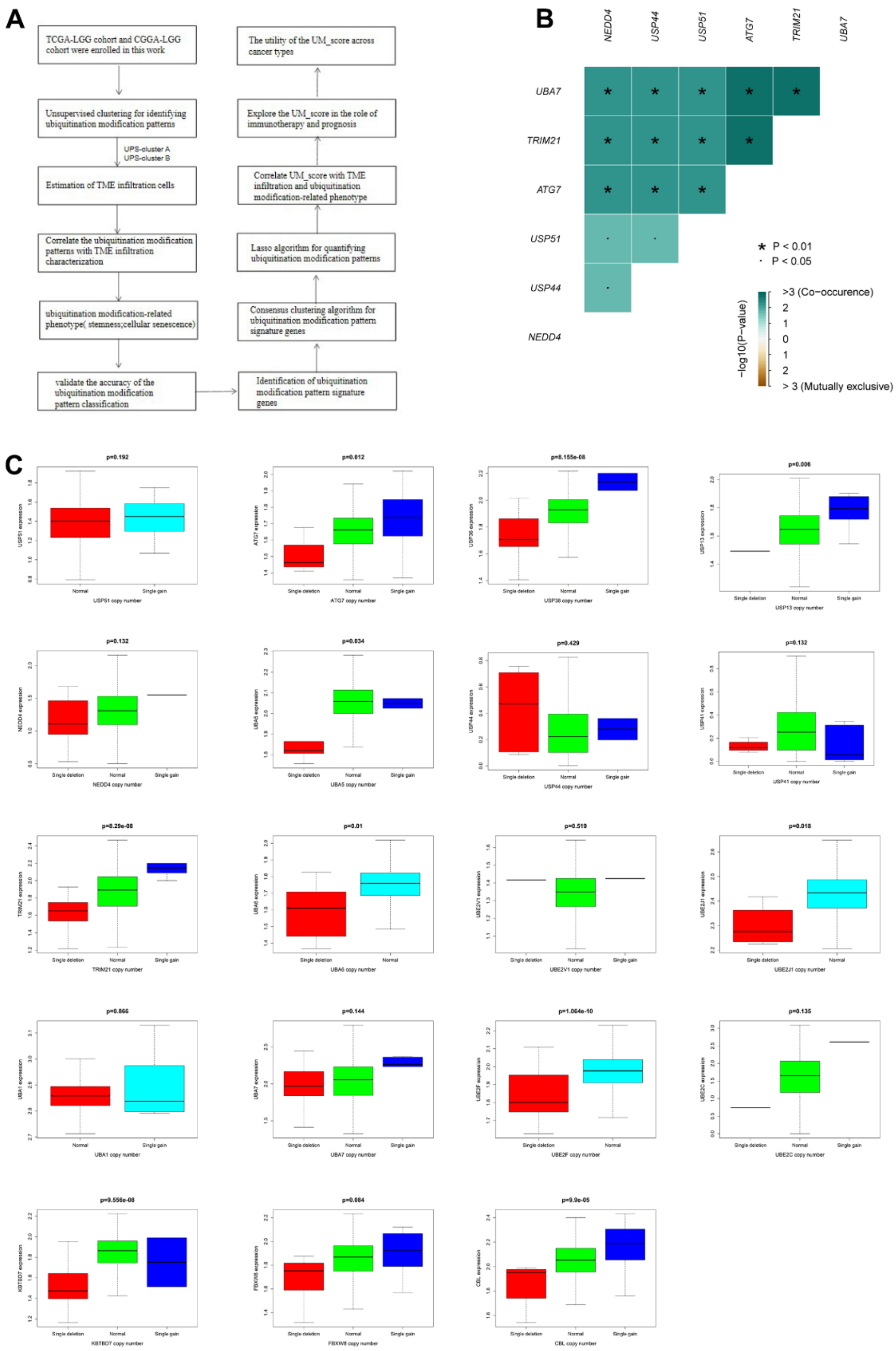

Supplementary Figure 1. The expression characteristic of ubiquitination modification regulators. (**A**) The entire design of this research; (**B**) Mutation co-occurrence correlation between UBA7 and ATG7, as well as TRIM21 and ATG7, along with TRIM21 and UBA7; (**C**) The correlation between ubiquitination regulator mRNA expression levels and CNV.

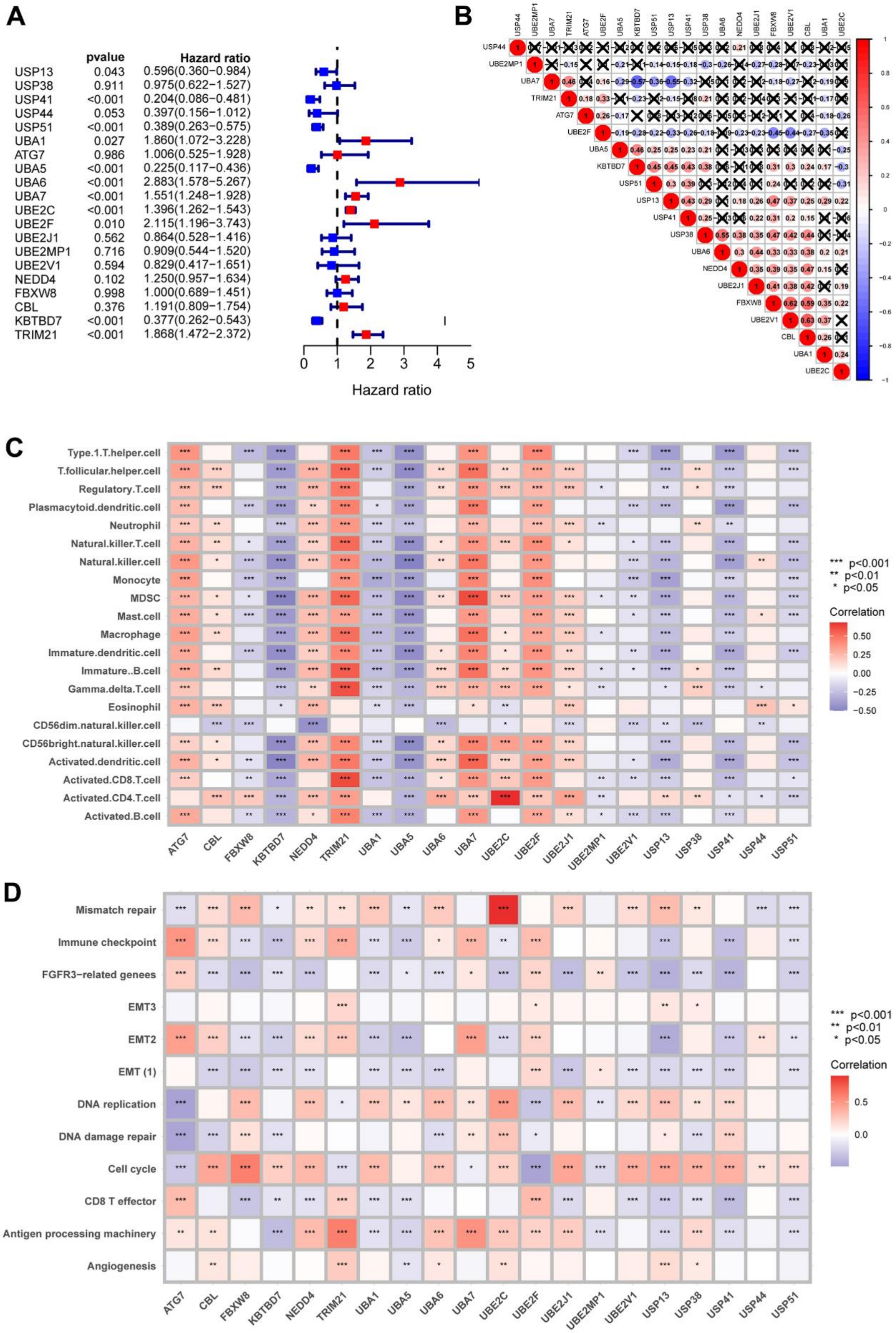

Supplementary Figure 2. The relationship between ubiquitination modification regulators and tumor microenvironment. (**A**) The prognostic values of 20 ubiquitination regulators; (**B**) Connections between the expression of 20 ubiquitination regulators in LGG; (**C**, **D**) The ubiquitination regulators were significantly linked to immune cell infiltration and biological processes related to the regulation of the tumor microenvironment.

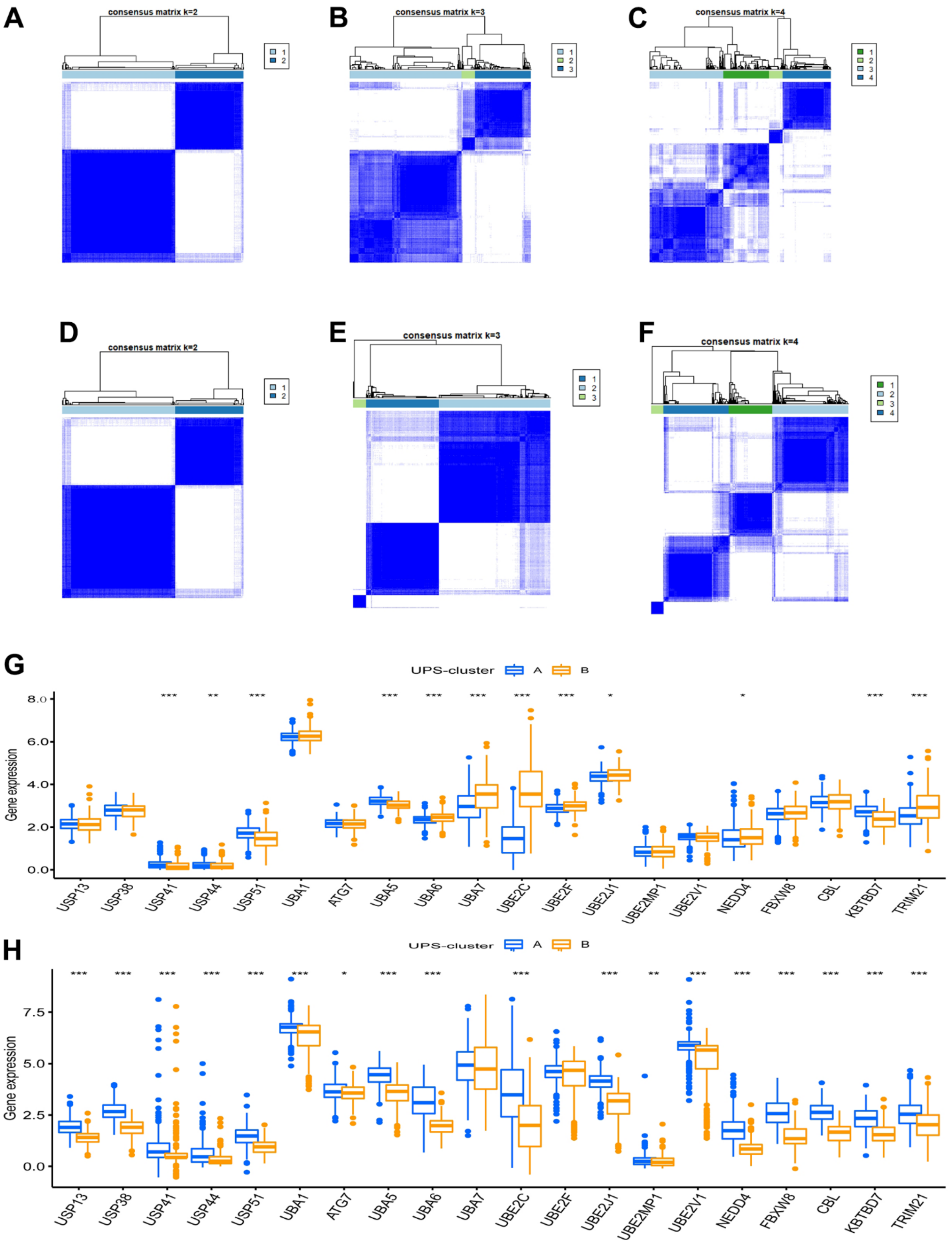

Supplementary Figure 3. The ubiquitination modification patterns in patients with LGG. (**A**–**C**) The ubiquitination modification patterns of LGG in TCGA; (**D**–**F**) The ubiquitination modification patterns of LGG in CGGA; (**G**, **H**) The distinction in transcriptome expression patterns of ubiquitination modification regulators between the USP-clusters in the TCGA-LGG and CGGA-LGG cohorts.

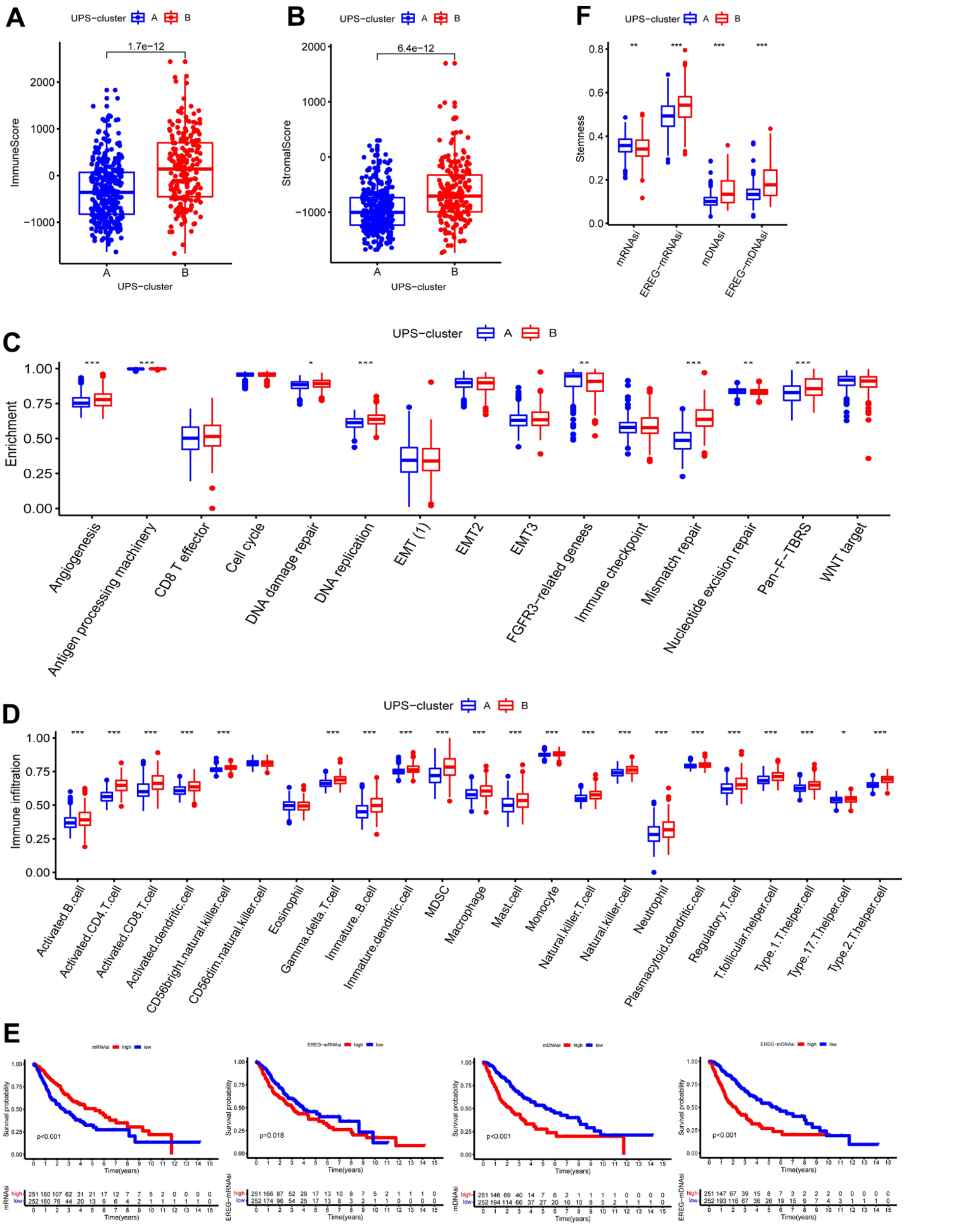

Supplementary Figure 4. Description of the TME in the two ubiquitination modification patterns. (**A**, **B**) The immune and stromal scores of the UPS-clusters; (**C**) The biological processes of the UPS-clusters; (**D**) The pro- and anti-tumor immune signatures of the UPS-clusters; (**E**) The stemness of cancer stem cells is significantly linked to the prognosis of LGG patients; (**F**) The stemness phenotype of the UPS-clusters.

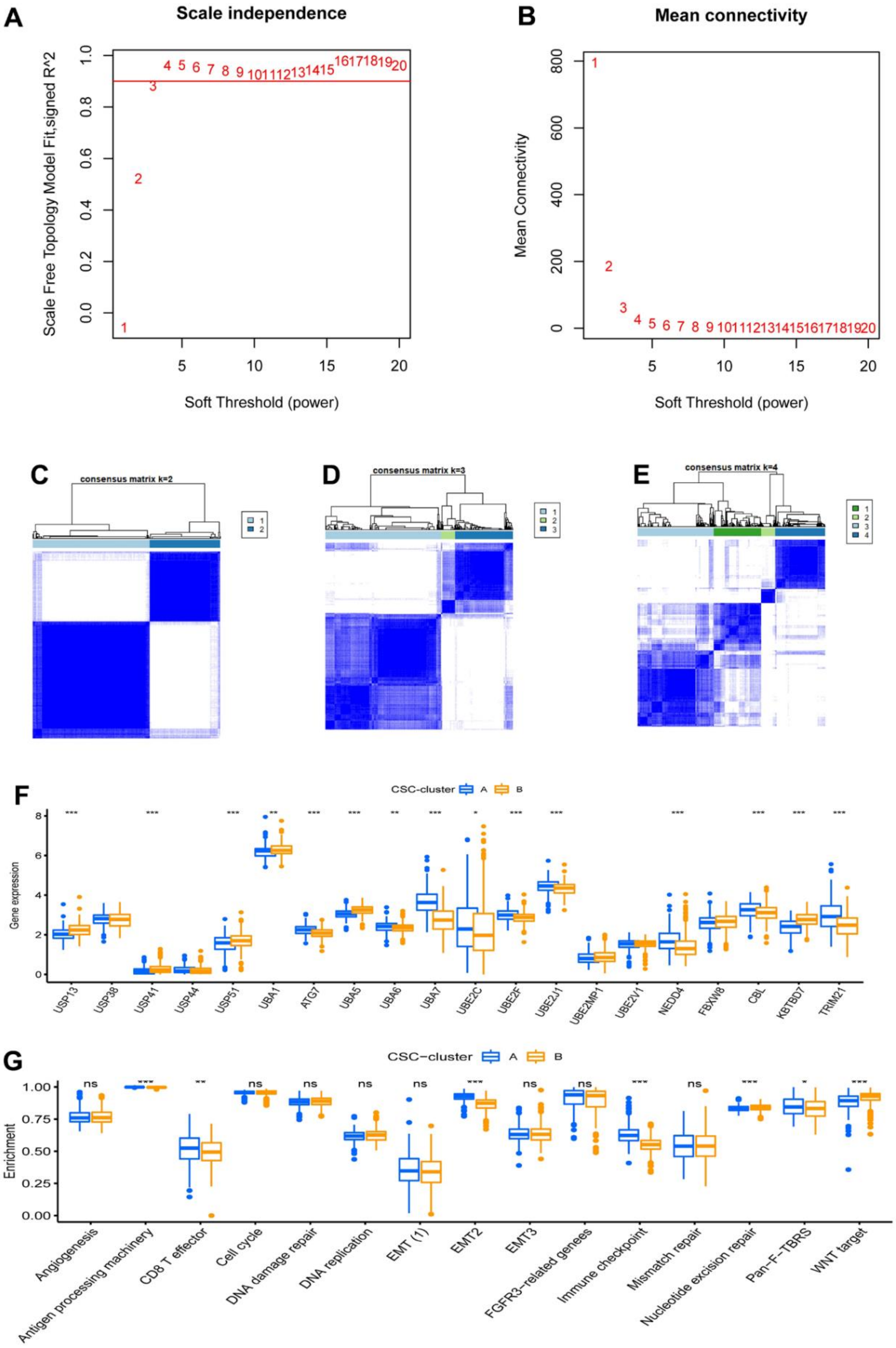

Supplementary Figure 5. The expression pattern of ubiquitination modification regulators between CSC-clusters. (**A**, **B**) The best parameter for transforming the adjacency matrix into a scale-free topology; (**C**–**E**) The distinct stem cell phenotypes of LGG patients; (**F**) The expression patterns of ubiquitination modification regulators in the two stem cell clusters; (**G**) The level of immune pathways of CSC-clusters.

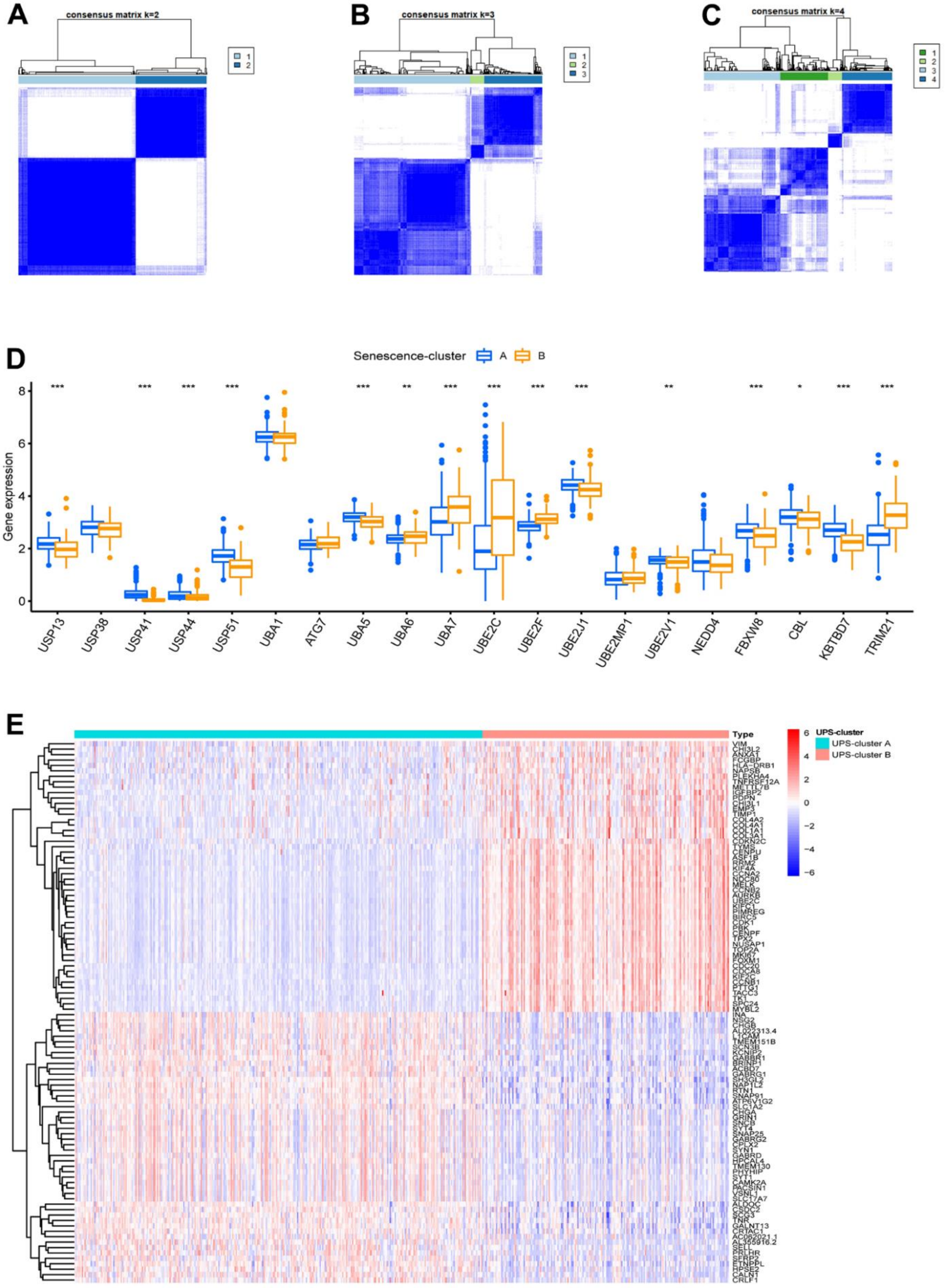

Supplementary Figure 6. The expression pattern of ubiquitination modification regulators between Senescence-clusters. (**A**–**C**) The distinct cellular senescence phenotypes of LGG patients; (**D**) The expression patterns of ubiquitination modification regulators in the senescence clusters; (**E**) The DEGs linked to the ubiquitination modification mode.

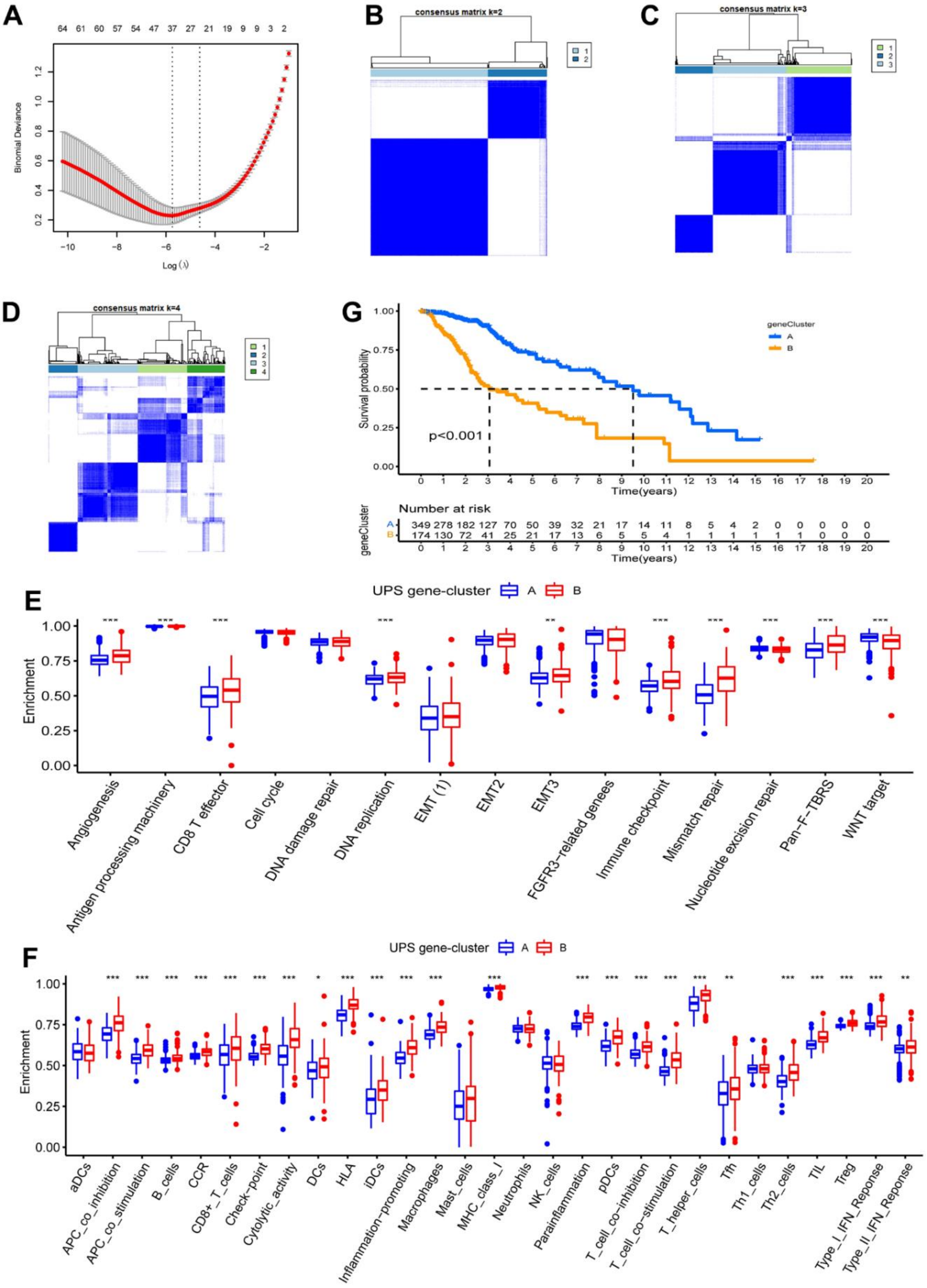

Supplementary Figure 7. Description of the TME in the two gene-clusters. (**A**) Lasso method on 216 DEGs to gain 37 signature genes of ubiquitination modification mode; (**B**–**D**) The UPS gene clusters of LGG patients; (**E**) The different level of stromal activity between the two gene-clusters; (**F**) The different pro- and anti-tumor immune signatures between the two gene-clusters; (**G**) The distinct prognosis between the two gene-clusters.

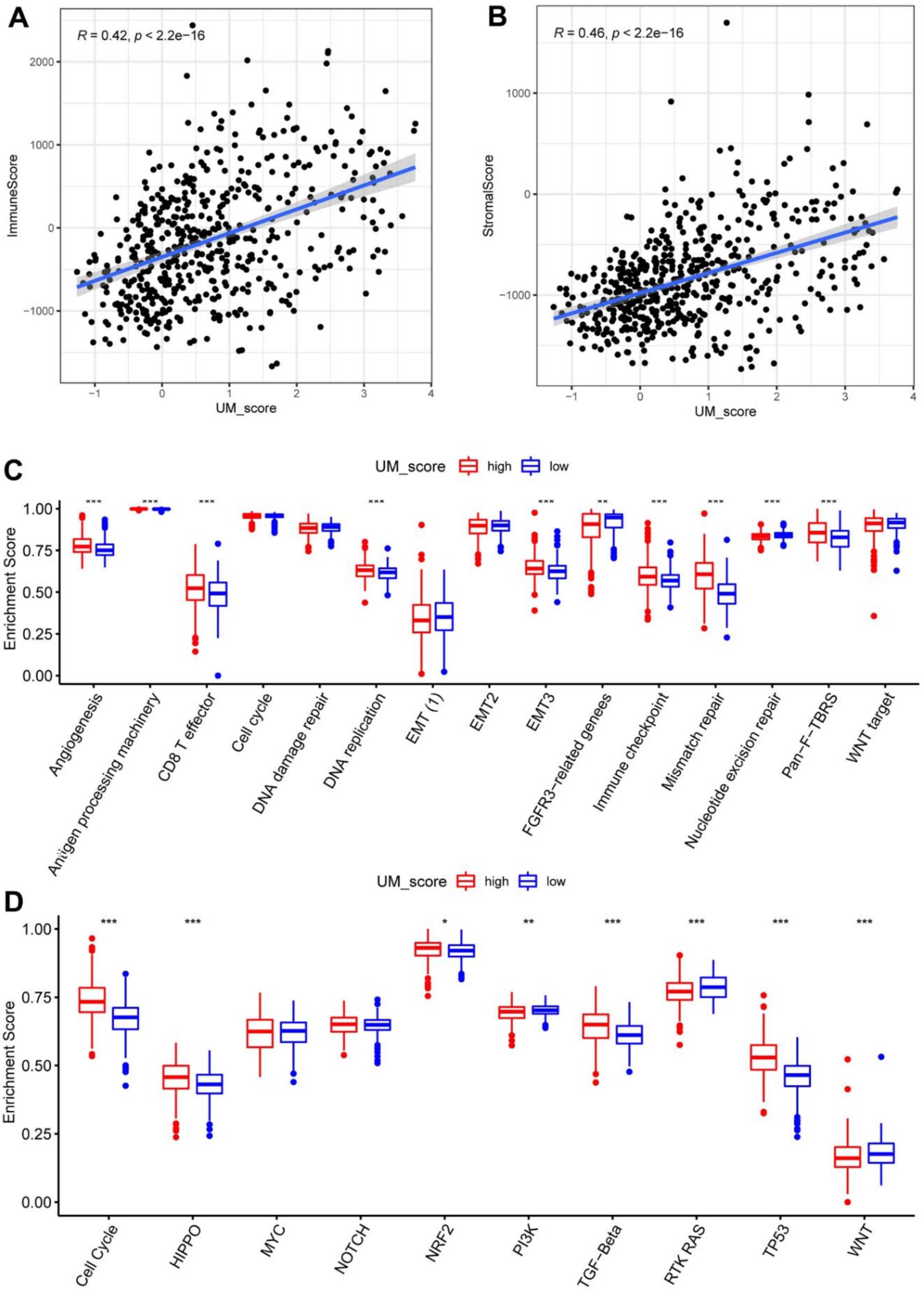

Supplementary Figure 8. Description of the TME in the two UM-score groups. (**A**, **B**) The UM-score was considerably positively linked to immune scores as well as stroma scores; (**C**) The different level of stromal-related biological processes between the two UM-score groups; (**D**) The different level of Fibroblast Growth Factor Receptor 3 (FGFR3), the RTK/RAS pathway, and the PI3K pathway between the two UM-score groups.

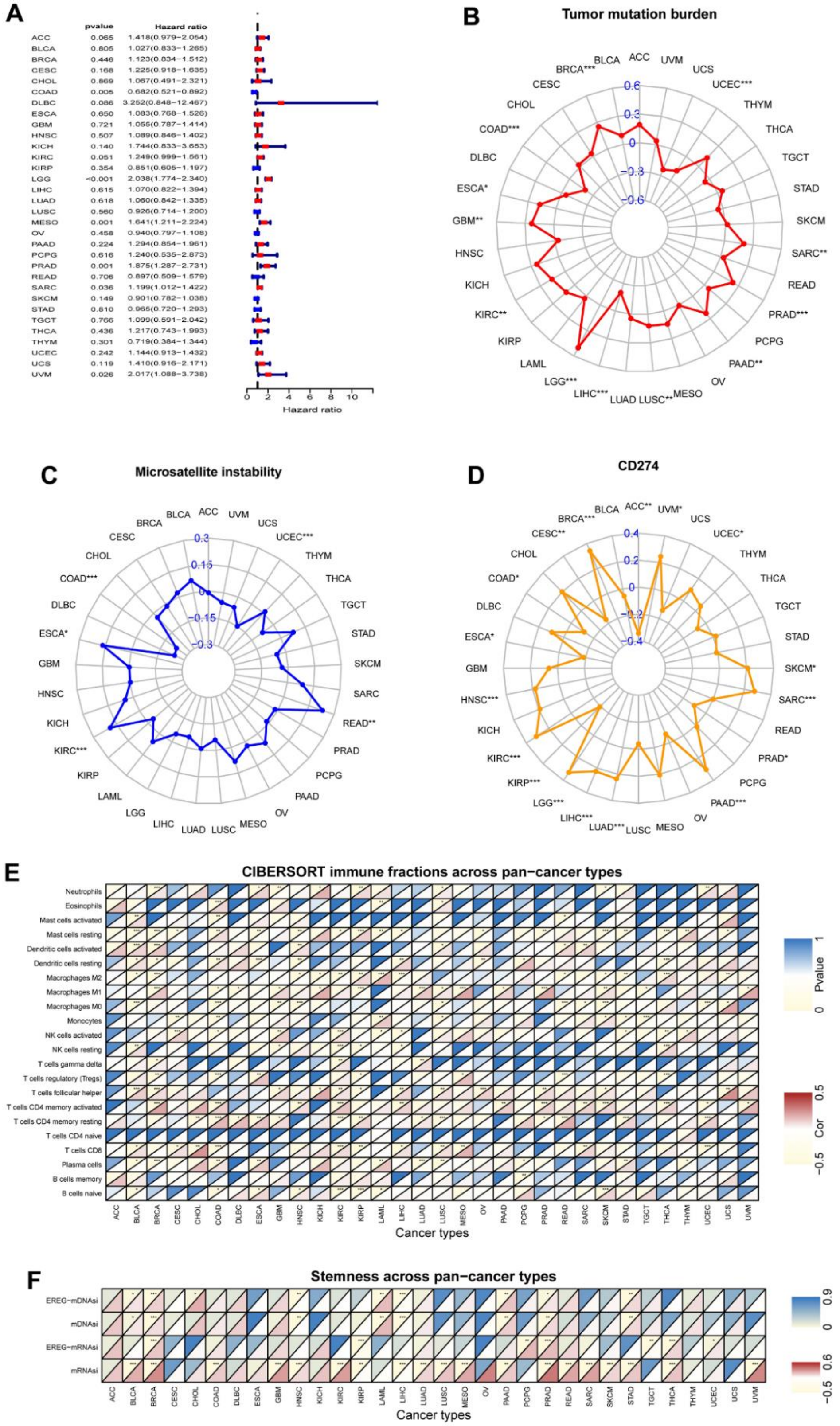

Supplementary Figure 9. The utility of the UM-score across cancer types. (**A**) The UM-score was linked to the prognosis of multiple kinds of cancer; (**B**) Radar plots revealed a significant correlation between UM-score and TMB in 12 of 33 cancers; (**C**) The correlation between MSI and UM-score; (**D**) The levels of PD-L1 expression were significantly related to the UM-score; (**E**) The ratio of M1 to M2 macrophages correlated with the UM-score of the majority of cancer types; (**F**) There is a link between UM-score and stemness index in 24 cancers.
