# Supplementary material for: Ubiquitin-proteasome system-mediated ubiquitination modification patterns and characterization of tumor microenvironment infiltration, stemness and cellular senescence in low-grade glioma
Source: Aging (Albany NY). 2023 Apr 11;15(8):2970–98. doi: 10.18632/aging.204650 (PMC10188348; doi:10.18632/aging.204650)
Supplement: Supplementary Tables 2 and 3 [file aging-15-204650-s003.pdf]

Supplementary Table 2. Gene list of ubiquitination modifications mode signature genes and independent prognosis-related signature genes.

Ubiquitination modifications mode signature genes	Independent prognosis-related signature genes
UBE2C	SFRP2
CDC20	FSTL1
AURKB	TACC3
TACC3	CD248
CDT1	CDT1
RTN1	UBE2C
SERPINH1	NAPSB
SCD	
TSPYL2	
MMP2	
SNAP91	
CD248	
FSTL1	
FRRS1L	
ACBD7	
JPH4	
KCNIP2	
NAPSB	
GALNT13	
APOL4	
HPCAL4	
COL6A2	
NRSN1	
INA	
FCGR3A	
ISG15	
VSTM2A	
VIM	
IFI6	
CSDC2	
GRIN1	
`HLA-DPA1`	
SFRP2	
F5	
FAM163B	
USH1C	
SLC14A1	

Supplementary Table 3. Genes used to construct the score and their coefficients.

Gene	Coef
SFRP2	-0.237798676450908
FSTL1	0.511913277444825
CD248	-0.318666391623948
CDT1	-0.323570756788893
UBE2C	0.394035278695233
NAPSB	0.14550177126947
